# Baseline Mood and “Relational Triad” Predict Acute Qualities of Psychedelic Experience

**DOI:** 10.3390/bs16020310

**Published:** 2026-02-23

**Authors:** Joshua Lipson, Hannes Kettner, Robin Carhart-Harris, Lisa Miller

**Affiliations:** 1 Center for Psychedelics and Spirituality, Emory University, Atlanta, GA 30322, USA; 2 Spirituality Mind Body Institute, Department of Clinical Psychology, Teachers College, Columbia University, New York, NY 10027, USA; drlisamiller@gmail.com; 3 Department of Neurology, University of California San Francisco, San Francisco, CA 94143, USA; hannes.kettner@ucsf.edu; 4 School of Medicine, University of California San Francisco, San Francisco, CA 94143, USA; robin.carhart-harris@ucsf.edu

**Keywords:** psychedelics, mysticism, spirituality, mindfulness

## Abstract

Background: The quality and valence of psychedelic experiences are influenced by a range of psychological and contextual factors. This study examines baseline mood and the “relational triad”—comprising social connectedness, mindfulness, and spirituality—as potential predictors of the quality of naturalistic psychedelic experiences. Methods: Data were drawn from the Predicting Responses to Psychedelics dataset, a longitudinal study tracking 654 individuals planning to take a psychedelic substance. Participants completed self-report measures at five time points, before and after ingestion. Baseline mood (depression, anxiety, and wellbeing) and relational triad factors were assessed at Timepoint 1, while acute psychedelic experience quality was measured at Timepoint 3 using validated scales (MEQ-30, CEQ, and ASC). Results: Mystical and challenging experiences were weakly but positively correlated. Baseline depression and anxiety were predictive of more challenging experiences but not of mystical-type experiences, while baseline wellbeing predicted more mystical and less challenging experiences. Mindfulness and spirituality were positively associated with mystical experiences, while social connectedness and mindfulness were inversely associated with challenging experiences. Conclusions: These findings extend previous research by demonstrating that baseline psychological and relational factors shape the nature of psychedelic experiences.

## 1. Introduction

Since the mid-20th century, psychedelics (a coinage based on the Greek *psyche*, mind + *delos*, manifesting) have captured the interest of research scientists, psychotherapists, spiritual seekers, and artists in the West (Osmond, 1957). Decades after a crackdown on recreational use and subsequent closure of research into psychedelics in the late 1960s and early 1970s, investigations into the mechanisms, benefits, and potential risks of psychedelics have entered a veritable “renaissance”—with a focus on the potential of psychedelic compounds’ power to treat depression, end-of-life anxiety, addiction, traumatic disorders, and other maladies (Griffiths et al., 2016; Reiff et al., 2020). On a molecular neurochemical level, classic psychedelics are a family of substances with substantial agonist effects at the 5-HT_2A_ serotonin receptor subtype, including a range of naturally occurring and synthetic drugs such as psilocybin, DMT/ayahuasca, bufo/5-MeO-DMT (tryptamines), LSD (a lysergamide), and mescaline/peyote (a phenethylamide) (Carhart-Harris & Nutt, 2017; Canal, 2018). These compounds are known for producing dramatic and varied alterations in visual perception, sense of time, salience, and mood, effects which have been attributed to psychedelics’ induction of increased suggestibility, suppression of default mode network activity, and dampening of top-down neural priors/promotion of entropy (Carhart-Harris et al., 2015; Preller et al., 2017; Hartogsohn, 2018; Palhano-Fontes et al., 2015; Carhart-Harris & Friston, 2019).

While it is possible to study the mechanisms and effects of psychedelics at many different levels of analysis, a great deal of psychedelic research has focused on the quality of the subjective, time-bound *experience* produced when these substances are ingested. Uniquely among drug classes, psychedelics produce a state of consciousness often experienced as especially *meaningful*, which have been shown to often qualify as mystical experiences, according to criteria that have come to be considered classic in modern Western research and theory (Hartogsohn, 2018; Plesa & Petranker, 2023). As such, despite arguments to the contrary (Olson, 2020), a preponderance of evidence suggests that psychedelics cannot be understood simply as a function of their pharmacological properties and neural correlates but rather as agents that produce *an experience* (Yaden & Griffiths, 2020; Ko et al., 2022).

The present study centers around the basic research question: what determines the valence and quality of the experience someone has any given time he or she ingests a psychedelic drug? While the relationship between a pharmacological agent and a target outcome is often modeled in broadly mechanistic terms, it is equally important to study effects that are mediated by inner states and experienced idiosyncratically, with particular attention to interindividual heterogeneity. In the case of psychedelics—substances long noted for their capacity to occasion experiences that range from profoundly challenging to deeply positive—it is therefore as vital to characterize the distribution and range of subjective experiences, as well as the factors associated with them, as it is to identify central tendencies or average effects.

In this study, we aim to contribute to the state of knowledge by (1) characterizing the relationship between different aspects of experience (e.g., “mystical” and “challenging”) in novel ways, (2) focusing on the roles of baseline mood and a set of psychosocial/psychospiritual factors which we call the “relational triad” (Lipson et al., 2024), and (3) situating our investigation in a *naturalistic* (rather than clinical) and *longitudinal* (rather than cross-sectional) context.

The present investigation is theoretically grounded in the literature on relational spirituality and, namely, in the “relational triad” framework developed by Lipson et al. (2024). In Lipson et al. (2024), the authors identified a distinctive set of factors, all implicated in relational spirituality, which were found to moderate the relationship between mystical experience and measures of mental health/psychopathology. Namely, the authors found that for individuals higher in belongingness, mindfulness, and spirituality (understood in terms of a “transcendent relationship”), mystical experience was associated with lower levels of psychopathology, while for individuals lower in these three factors, mystical experience was associated with higher levels of psychopathology. These analyses, however, were not particularly focused on *psychedelic-mediated* mystical experiences and relied on data collected cross-sectionally. This study aims to extend and replicate the cardinal findings of Lipson et al. (2024) in the specific context of psychedelic-mediated mystical experiences, with the benefit of temporal precedence that allows for the relationship between mystical experience and mental health to be modeled over time.

In order to undertake these analyses, we draw upon the “Predicting responses to psychedelics” dataset collected by Dr. Robin Carhart-Harris and his collaborators at Imperial College London, which served as the basis for the first published study of psychedelic experiences, their antecedents, and their effects that is both (1) focused on ingestion of psychedelics in naturalistic contexts and (2) longitudinal in design, comprising self-report data from five distinct timepoints before and after a psychedelic experience. This seminal study identified the roles of trait-level absorption, intention, set, setting, and dose in predicting the quality and valence of psychedelic experiences among an international sample (*n* = 654) of individuals planning to take a psychedelic in the near-term future (Haijen et al., 2018). Further studies based on the same sample conceptualized “emotional breakthrough” as a key component in psychedelic experiences and described the characteristics of attrition across the study’s course (Roseman et al., 2019; Hübner et al., 2021).

By bringing together the theoretical framework formulated in Lipson et al. (2024) and the Imperial College London dataset first published in Haijen et al. (2018), we propose to test a set of research hypotheses about the quality and predictors of naturalistic psychedelic experiences.

First, we aim to better characterize the relationship between mystical and challenging aspects of psychedelic experience. Haijen et al. (2018) identified drug dose and trait-level absorption as positive predictors of both mystical-type and challenging experiences but by contrast, identified different types of intention as predictive of more mystical or more challenging experiences, respectively. We seek to map the relative distribution of mystical-type and challenging experiences throughout our sample, as well as the nature of their association with one another. We hypothesize that mystical-type and challenging experience are *not* inversely associated with one another and may in fact be positively correlated with one another.

Second, we aim to better describe the relationship between baseline mood and the quality of psychedelic experience. Lipson et al. (2024) identified positive relationships between depression, anxiety, and wellbeing, on the one hand, and mystical experience, on the other. However, the role of baseline mood/wellbeing as a predictor of the quality and valence of naturalistic psychedelic experience remains poorly characterized. Given the differential, non-monotonic relationship between mystical experience and psychopathology uncovered in the latent profile analysis of Lipson et al. (2024), we predict that, overall, baseline depression, anxiety, and wellbeing will not be predictive of mystical experience one way or another. Moreover, we predict that baseline depression and anxiety will predict more challenging psychedelic experiences, while baseline wellbeing will predict less challenging psychedelic experiences.

Third and finally, we aim to better understand the relationship between the three components of the relational triad (Lipson et al., 2024)—belongingness/social connectedness, mindfulness, and spirituality—on the one hand, and the quality of a given psychedelic experience, on the other. Following the cross-sectional, non-psychedelic-specific findings of the former study, we predict that all three factors of the relational triad will be implicated in the types of experiences produced when people take a psychedelic. We also predict that relational triad factors will be predictive of less challenging experiences.

## 2. Materials and Methods

### 2.1. Participants and Procedure

The participant recruitment and demographics, as well as the study procedure, are exactly as detailed in Haijen et al. (2018). Individuals were considered to be eligible if they were planning to have an experience with one of the following classic psychedelic drugs: psilocybin/magic mushrooms/truffles, LSD/1P-LSD, ayahuasca, DMT/5-MeO-DMT, salvia divinorum, mescaline, or iboga/ibogaine. Data collected at Imperial College London by Carhart-Harris and Haijen under the protocol “Predicting acute- and long-term psychological effects in psychedelic drug users” were obtained through personal correspondence and invitation by the former. For further analysis of these previously collected data, ethics and consent exemption were then obtained through the Teachers College Institutional Review Board on 28 February 2024, under protocol #24-234.

The study procedure consisted of five surveys completed at different, pre-arranged moments. Participants completed the first survey one week before an anticipated psychedelic experience (Timepoint 1). Following this, participants completed the second survey one day before the planned psychedelic experience (Timepoint 2) and completed the third survey one day after the planned psychedelic experience (Timepoint 3). Participants then completed the fourth and fifth survey batteries two and four weeks after the psychedelic experience, respectively (Timepoint 4 and 5). An array of trait-level measures were taken repeatedly at Timepoint 1, 4, and 5 while Timepoint 2 and 3 engaged more directly with state-level measures of expectancy, set and setting, and acute experience (detailed below in the Method section).

The present study included 654 participants at Timepoint 1, 535 participants at Timepoint 2, 379 participants at Timepoint 3, 315 participants at Timepoint 4, and 212 participants at Timepoint 5.

**Figure 1:**
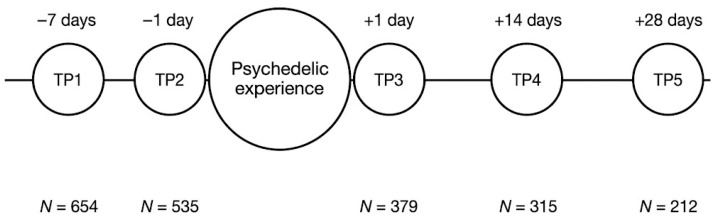
Procedure and sample sizes at relevant timepoints note: modified version of a figure originally published in Haijen et al. (2018).

A graphical overview of the procedure and of sample sizes at each timepoint is included in Figure 1 below, first printed in Haijen et al. (2018).

### 2.2. Key Measures

The present study draws on measures described in Haijen et al. (2018), with a focus on the following scales:

Mystical Experience. To measure mystical experience, the present study uses the revised, 30-item Mystical Experience Questionnaire (MEQ) validated by Barrett et al. (2015) in the context of psychedelic-occasioned mystical experiences. This most recent adaptation of the MEQ contains four validated subscales: “mystical” (example item: “Feeling that you experienced eternity and infinity”), “positive mood” (ex. “Feelings of tenderness or gentleness”), “transcendence of space and time” (ex. “Sense of being ‘outside of’ time, beyond past and future”), and “ineffability” (ex. “Sense that the experience cannot be described adequately in words”).

Challenging Experience. To measure unpleasant, challenging, or adverse aspects of acute psychedelic experiences, the present study uses the 26-item Challenging Experience Questionnaire developed by Barrett et al. (2016). Confirmatory factor analyses conducted during the initial validation identified seven distinct factors: fear (example item: “Frightened”), grief (ex. “Feelings of despair”), physical distress (ex. “Feel body shake/tremble”), insanity (ex. “Fear that you might lose your mind or go insane”), isolation (ex. “I felt isolated from everything and everyone”), death (ex. “Profound experience of your own death”), and paranoia (ex. “Feeling that people were plotting against you”).

Altered States of Consciousness. To measure a wide range of alterations in the phenomenology of consciousness experienced while under the influence of psychedelics, the present study uses the 42-item Altered States of Consciousness questionnaire developed by Studerus et al. (2010) out of its antecedent, the OAV (Bodmer et al., 1994), with the goal of identifying a stable factorial structure across different modes of altered consciousness induction modes. Factor analyses conducted on pooled response data from 43 experimental studies with psyilocybin, ketamine, and MDMA yielded eleven distinct factors: experience of unity, spiritual experience, blissful state, insightfulness, disembodiment, impaired control and cognition, anxiety, complex imagery, elementary imagery, audiovisual synaesthesia, and changed meaning of percepts.

Depressive Symptoms. The present study uses the Quick Inventory of Depressive Symptomatology (QIDS), a 16-item self-rated measure developed by Rush et al. (2003), to capture severity of cognitive, affective, and somatic depressive symptoms.

Anxiety Symptoms. The present study uses the Spielberger State-Trait Anxiety Inventory (STAI). Developed by Spielberger as a self-report measure, this scale is designed to capture both state- and trait-level anxiety, each with their own set of anxiety-relevant statements (Spielberger, 1983). The present study uses the portion of the STAI aimed at capturing trait-level anxiety, the STAI-T, as measured at Timepoint 1.

Overall Wellbeing. The present study uses the Warwick-Edinburgh Mental Wellbeing Scale (WEMWBS), a 14-item scale developed to measure “hedonic and eudaimonic” psychological and emotional wellbeing across an array of dimensions, including positive affect, satisfying interpersonal relationships, and “positive functioning” (Tennant et al., 2007).

Social Connectedness. Corresponding to Lipson et al.’s (2024) use of the General Belongingness Scale (GBS), we draw upon Haijen et al.’s (2018) use of an abridged, eight-item form of the Social Connectedness Scale (SCS), a self-report questionnaire developed by Lee and Robbins (1995). This scale aims at measuring the degree to which individuals feel connected to others in their social environment, or, in the words of its authors, belongingness.

Mindfulness. Corresponding to Lipson et al.’s (2024) use of the Five Facet Mindfulness Questionnaire (FFMQ), the present study uses the Cognitive and Affective Mindfulness Scale-Revised, a broad-based measure of mindfulness designed by Feldman et al. (2007) to stand on its own from belief and meditation practice.

Spirituality. In the present study, we refer to Haijen et al.’s (2018) two scales chosen to proxy spirituality. The first is Spiritual and Religious Attitudes in Dealing with Illness (SpREUK), which shares with Delaney’s Spirituality Scale (used in Lipson et al., 2024) an inherently relational component. We follow Haijen et al.’s (2018) use of an abridged, six-item version of this scale, developed by Büssing et al. (2005), to proxy the spiritual and religious attitudes observed as relevant to coping through chronic disease.

The second is the Universality subscale of Piedmont’s (1999) Spiritual Transcendence Scale (STS), which shares with Delaney’s Spirituality Scale (used in Lipson et al., 2024) a sense of spirituality as transcendent relationship. This scale, which aims to measure “the capacity of individuals to stand outside of their immediate sense of time and place and to view life from a larger, more objective perspective” in a way that is common to all major spiritual traditions, includes a nine-item subscale focused on “a belief in the unitive nature of life” (Piedmont, 2001).

Absorption. The present study makes limited use of the Modified Tellegen Absorption Scale (MODTAS), a self-report measure designed to assess trait-level absorption, defined as a stable individual difference reflecting the tendency to become fully engaged in perceptual, imaginative, and experiential states (Tellegen & Atkinson, 1974; Jamieson, 2005). Absorption has been shown to predict the intensity and phenomenological richness of altered states of consciousness, including those induced by psychedelics, and has been identified as a robust predictor of both mystical-type and challenging psychedelic experiences in prior analyses of the present dataset (Haijen et al., 2018).

Dose. The present study used a five-point Likert scale to assess the drug dose that participants understood themselves to have taken to precipitate the psychedelic experience reported upon at Timepoint 3. Irrespective of the particular substance ingested, the scale points were anchored by 1, “a low dose (for example no more than half a tab/50 micrograms of LSD)” and 5, “an extremely high dose (for example, no more than three tabs/300 micrograms of LSD)”.

### 2.3. Demographics

Of the 654 participants who responded at Timepoint 1, the mean age was 28.9 (*SD*  =  10.4), 74.2% of whom self-identified as male. About half of participants reported their highest level of education as either a bachelor’s degree (29.5%) or a graduate degree (20.2%), and 39.1% reported that they were currently enrolled as students. Half of participants were from the U.S. (30.4%) or the U.K. (19.6%), followed by Denmark (9.2%), Germany (4.9%), and Canada (4.9%), among 50 countries represented in total.

Of the 379 participants who completed both the baseline (T1) and post-experience (T3) assessments, the mean age was 30.6 years (*SD* = 11.0) and 66.5% self-identified as male. Compared to the baseline-only responders, participants retained at follow-up were more highly educated: 57.0% held at least a bachelor’s degree (34.5% bachelor’s and 22.5% postgraduate). Approximately one-third of T1 + T3 participants (32.7%) reported current student status. Participants were primarily based in the United States (27.6%) and the United Kingdom (21.1%), followed by Denmark (12.0%), Germany (6.0%), and Canada (3.7%), with representation from over 40 countries in total.

### 2.4. Data Analyses

All of the analyses described below were conducted using IBM SPSS Statistics, version 29.0 (IBM Corp, 2023).

Sample-Wide Correlational Analyses. Pearson correlation indices were computed to determine the overall relationships between social connectedness, mindfulness, and spirituality at baseline (Timepoint 1), as well as the relationships between depression symptoms, anxiety, and wellbeing at baseline (Timepoint 1). Additionally, Pearson correlation indices were computed to determine the overall relationships between mystical-type experience, challenging experience, state-level anxiety, and dimensions of altered consciousness, all measured at Timepoint 3. Then, correlational indices were computed to determine the overall relationships between mood and wellbeing at baseline (Timepoint 1), on the one hand, and measures of the quality of acute experience at Timepoint 3, on the other. Similarly, correlational indices were computed to determine the overall relationships between relational triad factors (social connectedness, mindfulness, and spirituality) at baseline (Timepoint 1), on the one hand, and measures of the quality of acute experience at Timepoint 3, on the other.

Developing a Typology of Experience. A scatterplot of challenging experience (CEQ) by mystical experience (MEQ) scores was generated, with MEQ scores plotted along the *x*-axis, and CEQ scores plotted along the *y*-axis. The scatterplot was visually inspected, and both high-MEQ/low-CEQ and low-MEQ/high-CEQ groups were identified as being of analytical interest. The sample was binarized at the mean value for MEQ, as well as at the mean value for CEQ. Following these procedures, four groups were obtained: one that was above average in mystical experience and below average in challenging experience, a second that was below average in mystical experience and above average in challenging experience, one that comprised individuals high in relative levels of both mystical experience and challenging experience, and one that comprised individuals low in relative levels of both mystical and challenging experience.

Between-Groups Comparisons. Analyses of variance (ANOVA) were used to compare the three groups obtained by the aforementioned procedure in terms of baseline depression, anxiety, and wellbeing, as well as in terms of baseline social connectedness, mindfulness, and spirituality. First, an omnibus between-groups ANOVA procedure was performed to determine if the groups differed overall in terms of each of the selected continuous comparator variables. Second, pairwise comparisons were conducted using the least significant difference (LSD) method in order to determine whether significant differences existed between any given pair among the three groups along each of the selected continuous comparator variables.

## 3. Results

### 3.1. Descriptive Statistics

Descriptive statistics for all variables of interest are summarized in Table 1 and Table 2, including measures of relational triad variables at baseline (Timepoint 1), measures of mood and wellbeing at baseline (Timepoint 1), and measures of quality of acute psychedelic experience (Timepoint 3).

Notably, baseline psychological characteristics were broadly similar between participants who completed both baseline and post-experience assessments and the full baseline sample, with only small differences observed across measures of mood, wellbeing, mindfulness, and spirituality.

### 3.2. Baseline Correlations: Timepoint 1

First, correlational analyses were run among each of the two sets of variables hypothesized to predict quality of acute psychedelic experience. Simple correlations were performed to assess the baseline associations between the three variables that define the relational triad: social connectedness as measured by the Social Connectedness Scale (SCS), mindfulness as measured by the Cognitive and Affective Mindfulness Scale (CAMS-R), and spirituality as measured by the SpREUK (Spiritual and Religious Attitudes) and STS-Universality (Spiritual Transcendence Scale—Universality Subscale). The results are shown in Table 3.

The two baseline measures of spirituality included in the dataset, SpREUK and STS-Universality, though conceptually non-identical, were found to be strongly positively correlated with one another (*r* = 0.807, *p* < 0.01), suggesting strong convergent validity between the measures for the purposes of further analyses. As a result, we elected to compute a compound score reflecting individuals’ scores on both measures by converting individuals’ scores on the SpREUK and STS-Universality subscale to z-scores, summing them, and taking the average. Both measures of baseline spirituality were weakly but positively associated with baseline mindfulness (*r* = 0.198 and 0.229, *p* < 0.01) and weakly but positively associated with baseline social connectedness (*r* = 0.119 and 0.151, *p* < 0.01). Meanwhile, at baseline, mindfulness and social connectedness were found to be moderately positively correlated with one another (*r* = 0.427, *p* < 0.01).

Additional simple correlations were performed to assess the baseline associations between the three selected baseline measures of mood: depression symptoms (negatively coded), as measured by the Quick Inventory of Depression Symptoms (QIDS); trait-level anxiety (negatively coded), as measured by the State-Trait Anxiety Inventory—Trait Subscale (STAI-T); and wellbeing (positively coded), as measured by the Warwick-Edinburgh Mental Wellbeing Scale (WEMWBS). These results are detailed in Table 4.

At baseline, depression symptoms and trait-level anxiety were strongly positively associated with each other (*r* = 0.639, *p* < 0.01), while baseline wellbeing was strongly negatively associated with baseline measures of both depression symptoms (*r* = −0.638, *p* < 0.01) and trait-level anxiety (*r* = −0.697, *p* < 0.01).

### 3.3. Correlations at the Level of Acute Experience (Timepoint 3)

Second, correlational analyses were run among measures of acute psychedelic experience taken at Timepoint 3 in order to assess the relatedness or non-relatedness of different measures of the quality and phenomenology of the experience, including mystical experience, as measured by the Mystical Experience Questionnaire (MEQ); challenging experience, as measured by the Challenging Experience Questionnaire (CEQ); and the 11 facets of phenomenology of experience, as measured by the subscales of the Altered States of Consciousness Questionnaire (ASC). The results of these analyses are summarized in Table 5.

An examination of correlations among different measures of the quality of acute psychedelic experience taken at Timepoint 3 reveals a bevy of significant associations. Retrospectively recalled state-level anxiety immediately before the experience was not significantly correlated with mystical experience but was moderately positively correlated with challenging experience (*r* = 0.362, *p* < 0.01). Meanwhile, mystical experience and challenging experience were found to be weakly but positively correlated with one another (*r* = 0.287, *p* < 0.01), an association which remained significant, though mildly attenuated, when controlling for drug dose (*r* = 0.23, *p <* 0.01) and for drug dose plus absorption (*r =* 0.20, *p* < 0.01), the two factors identified by Haijen et al. (2018) as significant positive predictors of both MEQ and CEQ scores. Mystical experience was found to be positively associated with all facets of phenomenology of experience on the ASC, while challenging experience was positively associated with all but the Blissful State subscale, with which it was not significantly associated. State-level anxiety immediately prior to the experience was positively associated with the Impaired Cognition and Control and Anxiety subscales of the ASC and negatively associated with the Audiovisual Synaesthesia subscale.

### 3.4. Relational Triad and Baseline Mood as Predictors of Acute Experience

Third, correlational analyses were performed to assess for the role of relational triad variables (as measured at Timepoint 1) as predictors of the quality and phenomenology of acute experience (as measured at Timepoint 3). Results are summarized in Table 6.

Social connectedness at baseline was not found to be associated with mystical experience but was found to be inversely associated with challenging experience (*r* = −0.142, *p* < 0.01). In addition, social connectedness was found to be inversely correlated with Impaired Cognition and Control but was not significantly associated with any other subscales of the ASC.

Mindfulness at baseline was found to be positively associated with mystical experience (*r* = 0.118, *p* = 0.028), as well as inversely associated with challenging experience (*r* = −0.231, *p* < 0.01). Moreover, mindfulness was found to be positively associated with the Experience of Unity, Spiritual Experience, and Blissful State subscales of the ASC and inversely associated with the Impaired Cognition and Control and Anxiety subscales of the ASC.

Finally, spirituality at baseline, as measured by a compound of the SpREUK and the STS-Universality scales, was found to be positively associated with mystical experience (*r* = 0.285, *p* < 0.01) but not significantly associated with challenging experience. Additionally, baseline spirituality was positively associated with the Experience of Unity, Spiritual Experience, Blissful State, and Insightfulness subscales of the ASC. Factors defined as part of the relational triad (i.e., belonging/social connectedness, mindfulness, and spirituality) were found not to be significantly predictive of six of eleven categories on the Altered States of Consciousness Questionnaire: disembodiment, complex imagery, elementary imagery, audiovisual synaesthesia, and changed meaning of percepts.

In addition, correlational analyses were performed to assess for the role of baseline mood and wellbeing (as measured at Timepoint 1) as predictors of the quality and phenomenology of acute experience (as measured at Timepoint 3). The results are summarized in Table 7.

Baseline depressive symptoms were found not to be associated with mystical experience but were found to be positively associated with challenging experience (*r* = 0.154, *p* < 0.01). In addition, baseline depressive symptoms were found to be positively associated with the Impaired Cognition and Control and Anxiety subscales of the ASC.

Baseline trait anxiety was found not to be associated with mystical experience but was found to be positively associated with challenging experience (*r* = 0.215, *p* < 0.01). In addition, baseline trait anxiety was found to be positively associated with the Anxiety subscale of the ASC. Meanwhile, baseline wellbeing was found to be positively associated with mystical experience (*r* = 0.135, *p* = 0.012), as well as inversely associated with challenging experience (*r* = −0.192, *p* < 0.01).

Moreover, baseline wellbeing was positively associated with the Experience of Unity, Spiritual Experience, and Blissful State subscales of the ASC, and inversely associated with the Impaired Cognition and Control and Anxiety subscales of the ASC.

Baseline measures of mood, both negatively coded (i.e., depression and anxiety) and positively coded (wellbeing) were found not to be significantly predictive of six of eleven categories on the Altered States of Consciousness Questionnaire: insightfulness, disembodiment, complex imagery, elementary imagery, audiovisual synaesthesia, and changed meaning of percepts.

### 3.5. Robustness to Dose

To determine whether observed associations between baseline mood, wellbeing, and relational triad variables, on the one hand, and acute psychedelic experience, on the other, were attributable to pharmacological intensity, all primary correlational analyses were re-estimated as partial correlations controlling for self-reported total dose. Across predictors, controlling for dose did not alter the direction or substantive magnitude of observed associations. Baseline depression and trait anxiety remained positively associated with challenging experience but not mystical experience, while baseline wellbeing remained positively associated with mystical experience and inversely associated with challenging experience. Similarly, mindfulness and spirituality remained positively associated with mystical experience, and mindfulness and social connectedness remained inversely associated with challenging experience. No previously null associations became significant after dose control.

### 3.6. Relational Triad and Baseline Mood Are Associated with a Typology of Experience

**Figure 2:**
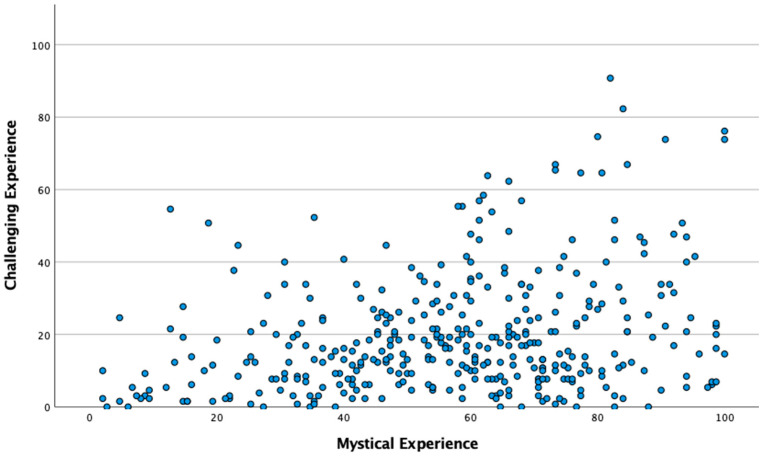
Scatter plot of challenging experience scores by mystical experience scores. Each blue dot represents a single sample individual’s MEQ (Mystical Experience Questionnaire) and CEQ (Challenging Experience Questionnaire) scores.

Following the finding that mystical and challenging experience are weakly but positively correlated with one another, a scatter plot was generated to examine the nature of this association and the degree to which it is monotonic and straightforwardly linear. A visual inspection of the scatter plot, shown in Figure 2, suggested that in the present sample, individuals high in mystical experience were often either high or low in challenging experience, while individuals low in mystical experience were most often also low in challenging experience. This pattern of association was maintained, though mildly attenuated, when controlling for drug dose and trait absorption.

In order to examine the predictors of different subtypes of psychedelic experience, in which levels of mystical and challenging experience may be either congruent or incongruent with one another (e.g., high/high, high/low), individuals who reported on the quality of their experience at Timepoint 3 were split into groups defined by either above-average or below-average levels of mystical experience, as well as above-average or below-average levels of challenging experience. This yielded two “incongruent” groups—a high mystical/low challenging group (*n* = 93) and a low mystical/high challenging group (*n* = 54)—as well as two “congruent” groups—a high mystical/high challenging group (*n* = 94) and a low mystical/low challenging group (*n* = 110). Analyses were run to determine whether baseline measures of mood and wellbeing, as well as baseline levels of relational triad factors (i.e. social connectedness, mindfulness, and spirituality), differed among these groups defined by Timepoint 3 quality of acute experience, as defined by levels of both mystical and challenging experience.

An omnibus comparison found significant differences among the four acute experience-defined groups in terms of baseline anxiety (*F* = 3.83, *p* = 0.010) and wellbeing (*F* = 4.06, *p* = 0.007) but not in terms of baseline depression (*F* = 0.927, *p* = 0.428). Notably, the group lowest in baseline anxiety was the *incongruent* high mystical/low challenging group; pairwise comparisons showed significant differences from both the *incongruent* low mystical/high challenging group (*p* = 0.014) and the *congruent* high mystical/high challenging group (*p* = 0.002). In terms of baseline wellbeing, the *incongruent* high mystical/low challenging group scored highest, while the *incongruent* low mystical/high challenging group scored lowest (*p* = 0.001). The two *congruent* groups, by contrast, were characterized by more intermediate average levels of baseline wellbeing. These results are summarized in Table 8.

Additionally, an omnibus comparison found significant differences among the four acute experience-defined groups in terms of baseline mindfulness (*F* = 6.59, *p* < 0.001) and spirituality (*F* = 10.85, *p* < 0.001) but not in terms of baseline social connectedness (*F* = 2.23, *p* = 0.085).

However, we note that the *incongruent* low mystical/high challenging group was characterized by the lowest baseline social connectedness scores—significantly lower than both the *incongruent* high mystical/low challenging group (*p* = 0.033) and the *congruent* low mystical/low challenging group (*p* = 0.039). Additionally, the groups scoring highest and lowest in average baseline levels of mindfulness were both characterized by *incongruent* levels of mystical and challenging experience: highest in baseline mindfulness was the high mystical/low challenging group, while lowest in baseline mindfulness was the low mystical/high challenging group (*p* < 0.001). The two MEQ-CEQ *congruent* groups were characterized by more intermediate levels of baseline mindfulness. Finally, while baseline spirituality appeared to primarily distinguish high-mystical groups from low-mystical groups, the *incongruent* high mystical/low challenging group was characterized by the highest baseline spirituality scores. These results are summarized in Table 9.

## 4. Discussion

The present study offers several findings that help to further describe the wide range of experiences that result from ingesting psychedelic substances in naturalistic, non-clinical contexts. Building upon the results reported by Haijen et al. (2018), we focused on the roles of baseline mood/wellbeing and the “relational triad” of social connectedness, mindfulness, and spirituality in predicting the quality and valence of psychedelic experiences. We note that, although many associations reached statistical significance, effect sizes were generally small. Accordingly, the findings discussed below should be interpreted as reflecting modest population-level tendencies rather than strong or individually predictive effects. Importantly, the full pattern of significant associations between baseline mood, relational triad factors, and experience quality remained in the same direction and statistically significant after adjustment for dose, a reliable predictor of generalized experience intensity.

### 4.1. Complete Mystical Experience Under Psychedelics

First, in a naturalistic, longitudinal sample of individuals, most of whom reported prior psychedelic experience, we found that just over a quarter (26.4%) of reported psychedelic experiences met the criteria for a “complete mystical experience”, defined by scoring at least 60% on all four primary indices of the mystical experience questionnaire (MEQ-30), i.e., mystical, positive mood, transcendence of time and space, and ineffability. This aligns closely with recently published data from a distinct, large-scale sample of naturalistic psychedelic experiences, which reports a 21.6% rate of “complete mystical experiences” (Nayak et al., 2023). While it is too early to say whether the present study and Nayak et al. (2023) have converged upon a natural law concerning the distribution of mysticism across psychedelic experiences, we suggest that future studies test against the benchmark of “about a quarter” of naturalistic psychedelic experiences producing complete mystical experiences. This rate, while impressive, contrasts with an emerging narrative, based on smaller, more carefully controlled experimental clinical studies, that psychedelics reliably or in even a majority of cases produce mystical experiences (Griffiths et al., 2006; Griffiths et al., 2011). It must be noted, however, that drug dose may be a significant confound in such comparisons between different samples and between naturalistic and experimental study designs, as considerable evidence suggests a strong association between dose and mystical experience (Haijen et al., 2018).

### 4.2. Relationships Among Qualities of Psychedelic Experience

We also found that, overall, experiences that were more mystical (as measured by the MEQ) also tended to be more challenging (as measured by the CEQ). Multiple studies have demonstrated that an array of manipulations, such as dosing with a psychedelic versus a placebo, as well as dosing in the context of SSRIs or not, tend to affect MEQ and CEQ scores in the same direction (Barbut Siva et al., 2024; Ko et al., 2023; Nayak et al., 2023; Sloshower et al., 2023). To some degree, this might be explained by shared factors or underpinnings that broadly predict the valence-neutral intensity of experience, such as trait-level absorption and drug dose (Haijen et al., 2018; Ko et al., 2023). However, the positive association between MEQ and CEQ scores remained statistically significant after adjusting for reported dose and trait absorption, suggesting that the co-occurrence of mystical and challenging features cannot be fully explained by generalized experience intensity alone.

However, we also found that, within the present sample, the association between mystical and challenging aspects of experience varied considerably, with substantial minorities of the sample reporting highly mystical but less challenging experiences, as well as highly challenging but less mystical experiences. This corresponds with emerging evidence of a typology of quality and valence of naturalistic psychedelic experiences, such as that reported by Nikolaidis et al. (2023). While Nikolaidis et al.’s (2023) approach, grounded in machine learning cluster analysis, identified high mystical/high challenging and low mystical/low challenging clusters, their analysis also revealed a distinctive high mystical/low challenging (“Positive”) cluster. Based on these findings, we suggest that it is important to spotlight the interaction between these two aspects of experience when modeling psychedelic trajectories—with a focus on whether levels of mystical and challenging experience are *congruent* or *incongruent*.

### 4.3. Baseline Mood and Wellbeing Are Associated with Quality of Psychedelic Experiences

**Figure 3:**
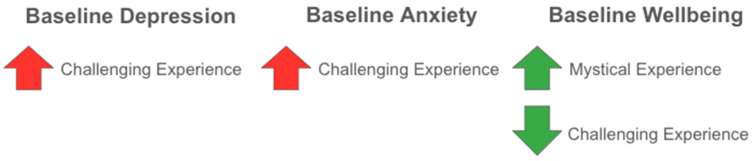
Baseline Mood-Level Predictors of Acute Experience.

Additionally, we found a distinct pattern of associations between mood and wellbeing at baseline, on the one hand, and the quality and valence of psychedelic experience, on the other. This pattern is summarized in Figure 3. Timepoint 1 depressive symptoms and baseline anxiety were not predictive of mystical experience at Timepoint 3, while Timepoint 1 wellbeing was weakly positively predictive of having a more mystical experience at Timepoint 3.

These results are of particular interest in light of the positive associations reported between mystical experience, on the one hand, and depression and anxiety, on the other, in Lipson et al. (2024), on which the conceptualization of the present study and its “relational triad” are based. When measured in a clear time series, baseline depression and anxiety were not associated with the degree of mystical experience two weeks later, following the ingestion of a psychedelic. Meanwhile, baseline wellbeing was weakly but positively associated with subsequent levels of mystical experience at Timepoint 3, echoing an association reported in Haijen et al. (2018). This pattern appears to correspond with Studerus et al.’s (2012) finding that baseline “anxiety-depressiveness” was generally not predictive of oceanic boundlessness (including constructs closely related to mystical experience) but that general wellbeing weakly predicted certain aspects of it. Ultimately, while depression and anxiety at baseline might have implications for whether psychedelic administration is advisable or likely to be clinically beneficial, it appears that they are not overall associated with the probability of having a mystical experience in one direction or the other.

By contrast, all three cardinal measures of baseline mood and wellbeing were significantly associated with higher or lower degrees of challenging experience at Timepoint 3. Baseline depression symptoms and trait-level anxiety, as measured at baseline, were weakly but positively associated with challenging experience, while baseline wellbeing was weakly but negatively associated with challenging experience. A similar pattern of associations, but with stronger magnitudes, was observed when focusing on state-level anxiety the day of the reported experience; baseline depression and anxiety were positively associated with it, while baseline wellbeing was negatively associated with it.

Given the robust extant literature attesting to the role of high state-level anxiety/negative mood in predicting challenging experiences, it is possible that high trait-level baseline measures of depression and anxiety are associated with high anxiety/negative mood states proximal to the experience, which may co-occur with more challenging experiences (Studerus et al., 2012). As such, individuals higher in baseline depression and anxiety intending to take a psychedelic in either a clinical or non-clinical setting may benefit from being informed that, on average, higher baseline depression and anxiety are associated with a greater likelihood of challenging experiences and prepared accordingly. This does not contradict evidence that psychedelics can be beneficial for these conditions under carefully controlled conditions, which typically include screening, preparation, therapeutic framing, and integration. It is also important to note that more challenging experiences do not necessarily generate measurable post-trip distress and may in some contexts even be followed by a decrease in anxious or depressive symptomatology.

Above and beyond their relationships to mystical and challenging features of experience, treated separately, baseline mood and wellbeing were found to be associated with distinct combinations of mystical and challenging features, such that individuals scoring high in mystical experience and low in challenging experience scored the lowest in baseline depression and anxiety and the highest in baseline wellbeing. Meanwhile, individuals scoring low in mystical experience and high in challenging experience scored highest in baseline depression and anxiety and lowest in wellbeing, while individuals scoring congruent levels of mystical and challenging experience fell in the middle of the range. This pattern suggests that baseline mood and wellbeing might be more powerfully predictive of distinct phenomenologically defined *types* of psychedelic experience than of either mystical or challenging experience alone. Nikolaidis et al. (2023) offer some evidence for this possibility, suggesting that individuals reporting experiences that are both less mystical and less challenging are on average lower in baseline depression and anxiety symptoms than *either* high-mystical/high-challenging *or* high-mystical/low-challenging scorers.

On a more phenomenologically granular level, baseline wellbeing weakly predicted greater levels of unity, spiritual experience, and bliss during the experience, as well as lower levels of impaired cognition and control and anxiety during the experience. Baseline anxiety and depression were both weakly predictive of greater anxiety during the experience, and depression was weakly positively associated with impaired cognition and control. In stark contrast, no measures of baseline mood and wellbeing were predictive of perceptual and somatosensory “primary process” aspects of psychedelic experience at Timepoint 3, such as complex and elementary imagery, synesthesia, and disembodiment. This suggests that different dimensions of psychedelic experience are differentially related to an individual’s affective set point and are potentially predicted by different sets of factors, a pattern similar to that observed in Studerus et al.’s (2012) analysis of the predictors of experience during experimental psilocybin sessions.

### 4.4. A Relational Triad Is Associated with Quality of Psychedelic Experiences

**Figure 4:**
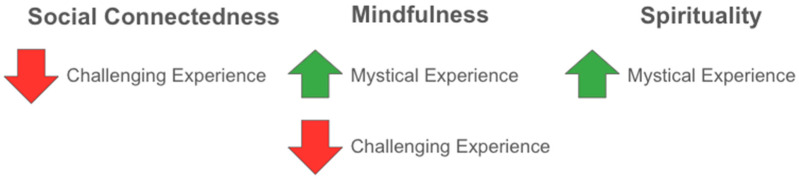
Baseline relational triad-level predictors of acute experience.

As hypothesized, all three factors comprising the relational triad, when measured at baseline, were found to be associated with the quality of the psychedelic experience reported at Timepoint 3. Namely, social connectedness was associated with lower levels of challenging experience, spirituality was associated with higher levels of mystical experience, and mindfulness was both associated with higher levels of mystical experience and lower levels of challenging experience. This pattern of results is summarized in Figure 4.

One possible interpretation is that higher social connectedness co-occurs with greater perceived grounding in the “default reality” of everyday consciousness, belonging to communities that offer sense-making beliefs and rituals and lower risk of alienation and interpersonal judgment. Of note, social connectedness was most distinctly associated with lower levels of isolation (*r* = −0.218) and paranoia (*r* = −0.190), aspects of the CEQ conceptualization of a challenging experience that can be identified as relational in nature.

The dual role of mindfulness as positively associated with mystical experience and associated with lower levels of challenging experience might be understood as reflecting the ability to relate to high-intensity, high-novelty states of consciousness with a greater degree of intentionality, control, and, when necessary, detachment. Notably, distinct sets of items from the Cognitive Affective Mindfulness Scale (CAMS-R) were associated most strongly with either more mystical or less challenging experience, respectively. In particular, higher degrees of mystical experience were predicted by a baseline ability to focus on the present moment, as well as by low distractibility, while lower levels of challenging experience were distinctly associated with ability to accept one’s thoughts and feelings and tolerate emotional pain.

Meanwhile, using conceptualizations of spirituality defined by a felt/perceived sense of reality as a unitive field and of a loving relationship with a higher power, individuals higher in baseline spirituality tended to report higher levels of mystical experience, a pattern consistent with interpretive frameworks emphasizing meaning-making around altered states consciousness. Meanwhile, the lack of any significant association between baseline spirituality and challenging experience might indicate (1) no particular association between spirituality and the valence of psychedelic experiences, or otherwise (2) an averaging of different sub-associations, with spirituality both protecting against general negative valence, while also predicting more metaphysically significant, and potentially more existentially disruptive experiences.

It is important to note, however, that mindfulness, spirituality, wellbeing, and mystical experience share substantial conceptual and phenomenological overlap. This overlap is not incidental but reflects long-standing theoretical traditions in which these constructs are understood as mutually entangled dimensions of human experience rather than as discrete psychological modules. As such, associations between baseline mindfulness or spirituality and subsequent mystical experience should not be interpreted as evidence of distinct or independent causal pathways.

As in the case of baseline mood and wellbeing, measures of all relational triad factors were found to be predictive of distinct combinations of mystical and challenging features, above and beyond their relationships to mystical and challenging features of experience, treated separately. Levels of baseline social connectedness were highest among individuals scoring high in mystical and low in challenging experience and lowest among individuals scoring low in mystical and high in challenging experience. In addition, mindfulness and spirituality scores were significantly higher among individuals high in mystical and low in challenging experience than among “mystical/challenging-congruent” individuals. Given the lack of observed association between social connectedness and mystical experience, as well as between spirituality and challenging experience, it appears that the best predictive use for relational triad factors (much like for baseline mood, as described above) is in relation to more complex, multidimensional typologies of psychedelic experience.

### 4.5. Limitations

The present study is also characterized by several limitations. First, given the scale of psychedelic use among U.S. and European adults, our sample size of 654 at Timepoint 1, and of 379 at Timepoint 3, is itself a primary limitation, and militates against over-generalization of our results to the broader population of non-clinical psychedelic users (Livne et al., 2022). Moreover, dropout from time point to time point over the course of the study represents a potential limitation to the validity of study findings, as a result of possible attrition bias. This subject has been discussed in detail with regard to the present sample, which found that demographic and personality characteristics, rather than quality of experience, were the factors most predictive of attrition (Hübner et al., 2021).

Second, inferences about the relationship between baseline mood and acute experience must be tempered by the understanding that “baseline mood”, as characterized in this study, refers to measurements taken a week before the psychedelic experience described. Given that the instrument used to measure depression in the present study refers to symptoms over the past week, it is possible that fluctuations in the week between Timepoint 1 (baseline) and Timepoint 3 (immediately following acute experience) may not be captured in the data. This may limit the reliability of these findings, though it may also lead to an underestimation of the magnitude of reported associations.

Third, although we conducted robustness analyses adjusting for dose (and trait absorption for analyses involving MEQ–CEQ associations), we did not adjust for additional contextual variables such as intention, setting, or prior psychedelic use. These variables are multi-dimensional and may plausibly function as mediators or moderators—rather than simple confounds—of the relationships between baseline psychological factors and acute experience in naturalistic designs. Accordingly, reported associations should not be interpreted as strictly independent effects, and future work using designs better suited to causal partitioning will be needed to clarify these pathways.

Fourth, we identify several sample-related limitations to these findings’ generalizability. First, the gender, racial, age, educational, and national background of the sample cannot be treated as representative of the general population. Nearly three-quarters of the sample was male, with a mean age of 28.9 (*SD* = 10.4), and a 39.1% share identifying as current students. Half of participants hailed from either the United States or the United Kingdom. While large-scale epidemiological data indicate higher levels of lifetime psychedelic use among males and whites, and a contemporary uptick in psychedelic use is reportedly concentrated among younger adults, the distinctive, non-representative demographic profile of the present sample requires any conclusions to be generalized to population-wise naturalistic use of psychedelics with great caution (Killion et al., 2021; Livne et al., 2022; Viña & Stephens, 2023).

Fifth, these findings reflect a sample likely shaped by self-selection bias, given that participant recruitment was partially conducted via psychedelic-themed social media pages, newsletters, and web forums. These online platforms disproportionately attract not only individuals who have used psychedelics but particularly those who take an active, passionate interest in them for cultural, therapeutic, or spiritual reasons. Relatedly, 91% of participants reported previous experience with psychedelics, with over half reporting six or more lifetime uses. The nature and quality of psychedelic experiences among those with considerable prior experience, as well as their relationship to baseline mood and relational triad factors, might not necessarily be generalizable to individuals’ first and second psychedelic experiences (which anecdotally are often reported to be more mystical and “profound”) (Studerus et al., 2010, 2012; McCartney et al., 2023).

### 4.6. Future Directions

The current study sets out an analytical structure aimed at identifying previously poorly characterized predictors of the quality and valence of psychedelic experience, as well as elucidating the relationship between mystical and challenging aspects of psychedelic experience. The analyses discussed above represent an effort to extend Haijen et al.’s (2018) “Predicting responses to psychedelics” project to incorporate baseline mood and the relational triad (belongingness/social connectedness, mindfulness, and spirituality). We suggest the present study’s analytic framework as an “out-of-the-box” framework for identifying additional simple predictors and moderators of the quality and valence of psychedelic experience.

Moreover, in the interest of testing whether the present study’s findings replicate more generally, we recommend that the present analytical structure, focused on the relationships between baseline mood, the relational triad, and quality/valence of psychedelic experience in a naturalistic context, be replicated in a larger, more representative sample. This would entail ensuring greater representation of women, racial minorities, and older individuals, as well as psychedelic-naïve participants.

Ultimately, we hope that the present study’s findings and analytic procedures can be integrated into a more comprehensive model of the “probability space” of psychedelic experiences, oriented toward mapping the probability space of psychedelic experiences, with the aim of supporting preparation, harm reduction, and contextual sensitivity rather than deterministic individual-level prediction. While much replication, generalization, and paradigm extension work will be necessary toward this end, the pending normalization of the clinical/therapeutic use of psychedelics—which anticipates a groundswell of use under a range of conditions, both supervised and unsupervised—raises the stakes for the development of models, tools, and protocols that admit to the wide range of experiences that psychedelics are capable of producing.

## Figures and Tables

**Table 1:** Baseline (TP1) measures of relational triad and mood for all TP1 responders.

Measure	Mean	SD	*n*
SCS	33.86	10.27	653
CAMS-R	32.48	5.65	654
SpREUK	17.44	6.60	654
STS	33.36	8.27	654
QIDS_1	6.28	4.38	654
STAI_T_1	12.07	3.81	654
WEMWBS_1	49.01	8.82	654

Key: SCS—Social Connectedness Scale, CAMS-R—Cognitive and Affective Mindfulness Scale-Revised, SpREUK—Spiritual and Religious Attitudes, STS-Universality—Spiritual Transcendence Scale (Universality Subscale), QIDS—Quick Inventory of Depressive Symptoms, STAI_T—State Trait Anxiety Inventory (Trait Subscale), WEMWBS—Warwick Edinburgh Mental Wellbeing Scale.

**Table 2:** Baseline (TP1) measures of relational triad and mood, and acute experience (TP3) for TP1–TP3 responders.

Measure	Mean	SD	*n*
SCS	34.11	10.01	350
CAMS-R	33.12	5.80	351
SpREUK	17.62	6.67	351
STS	33.75	8.27	351
QIDS_1	6.03	4.21	351
STAI_T_1	11.93	3.79	351
WEMWBS_1	49.62	8.74	351
Complete ME	26.4%		100
MEQ	57.07	22.63	379
CEQ	19.69	16.43	379
ASC-Unity	45.75	28.31	379
ASC-Spiritual	42.35	26.54	379
ASC-Blissful	57.79	30.42	379
ASC-Insight	48.65	26.88	379
ASC-Disembodied	27.75	25.58	379
ASC-Impaired	18.74	16.24	379
ASC-Anxiety	15.10	20.17	379
ASC-Complex Img	49.76	31.26	379
ASC-Elementary Img	60.35	32.09	379
ASC-Audiovisual	46.94	32.57	379
ASC-Changed Mean	45.34	27.40	379

Key: SCS—Social Connectedness Scale, CAMS-R—Cognitive and Affective Mindfulness Scale-Revised, SpREUK—Spiritual and Religious Attitudes, STS-Universality—Spiritual Transcendence Scale (Universality Subscale), QIDS—Quick Inventory of Depressive Symptoms, STAI_T—State Trait Anxiety Inventory (Trait Subscale), WEMWBS—Warwick Edinburgh Mental Wellbeing Scale, MEQ—Mystical Experience Questionnaire, CEQ—Challenging Experience Questionnaire, ASC—Altered Scales of Consciousness Questionnaire, with subscales: Unity—Experience of Unity, Spiritual—Spiritual Experience, Blissful—Blissful State, Insightful—Insightfulness, Impaired—Impaired Cognition and Control, Anxiety—Anxiety, Complex Img—Complex Imagery, Elementary Img—Elementary Imagery, Audiovisual—Audiovisual Synaesthesia, Changed Mean—Changed Meaning of Percepts.

**Table 3:** Correlations among relational triad variables at baseline (TP1).

	SCS	CAMS-R	SpREUK
CAMS-R	0.427 **		
SpREUK	0.119 **	0.198 **	
STS	0.151 **	0.229 **	0.807 **

Key: SCS—Social Connectedness Scale, CAMS-R—Cognitive and Affective Mindfulness Scale-Revised, SpREUK—Spiritual and Religious Attitudes, STS-Universality—Spiritual Transcendence Scale (Universality Subscale). ** *p* < 0.01.

**Table 4:** Correlations among measures of mood and wellbeing at baseline (TP1).

	QIDS_1	STAI_T_1
STAI_T_1	0.639 **	
WEMWBS_1	−0.638 **	−0.697 **

Key: QIDS_1—Quick Inventory of Depressive Symptoms (Timepoint 1), STAI-T_1—State-Trait Anxiety Inventory (Trait Subscale; Timepoint 1), WEMWBS_1—Warwick-Edinburgh Mental Wellbeing Scale (Timepoint 1). ** *p* < 0.01.

**Table 5:** Correlations among measures of acute experience (TP3).

	MEQ	CEQ
CEQ	0.287 **	
ASC-Experience of Unity	0.821 **	0.197 **
ASC-Spiritual Experience	0.717 **	0.209 **
ASC-Blissful State	0.719 **	−0.044
ASC-Insightfulness	0.659 **	0.160 **
ASC-Disembodiment	0.496 **	0.321 **
ASC-Impaired Cognition and Control	0.363 **	0.592 **
ASC-Anxiety	0.199 **	0.770 **
ASC-Complex Imagery	0.574 **	0.278 **
ASC-Elementary Imagery	0.518 **	0.240 **
ASC-Audiovisual Synaesthesia	0.465 **	0.200 **
ASC-Changed Meaning of Percepts	0.544 **	0.250 **

Key: MEQ—Mystical Experience Questionnaire, CEQ—Challenging Experience Questionnaire, ASC—Altered States of Consciousness Questionnaire. ** *p* < 0.01.

**Table 6:** Relational triad variables (TP1) as predictors of acute experience (TP3).

	SCS	CAMS-R	Spir_Comp
MEQ	−0.020	0.118 *	0.285 **
CEQ	−0.142 **	−0.231 **	0.058
ASC-Unity	0.023	0.151 **	0.298 **
ASC-Spiritual	0.035	0.165 **	0.412 **
ASC-Blissful	−0.009	0.178 **	0.171 **
ASC-Insightful	−0.056	0.088	0.151 **
ASC-Impaired	−0.107 *	−0.182 **	0.021
ASC-Anxiety	−0.090	−0.251 **	0.018

Key: SCS—Social Connectedness Scale, CAMS-R—Cognitive and Affective Mindfulness Scale-Revised, Spir_Comp—Spirituality Compound Score, MEQ—Mystical Experience Questionnaire, CEQ—Challenging Experience Questionnaire, ASC—Altered Scales of Consciousness Questionnaire, with subscales: Unity—Experience of Unity, Spiritual—Spiritual Experience, Blissful—Blissful State, Insightful—Insightfulness, Impaired—Impaired Cognition and Control. * *p* < 0.05, ** *p* < 0.01.

**Table 7:** Baseline measures of mood (TP1) as predictors of acute experience (TP3).

	QIDS_1	STAI_T_1	WEMWBS_1
MEQ	−0.006	−0.039	0.135 *
CEQ	0.154 **	0.215 **	−0.192 **
ASC-Unity	0.009	−0.045	0.137 *
ASC-Spiritual	−0.054	−0.061	0.169 **
ASC-Blissful	0.024	−0.063	0.152 **
ASC-Impaired	0.107 *	0.089	−0.126 *
ASC-Anxiety	0.122 *	0.179 **	−0.166 **

Key: QIDS_1—Quick Inventory of Depressive Symptoms (Timepoint 1), STAI-T_1—State-Trait Anxiety Inventory (Trait Subscale; Timepoint 1), WEMWBS_1—Warwick-Edinburgh Wellbeing Scale (Timepoint 1), MEQ—Mystical Experience Questionnaire, CEQ—Challenging Experience Questionnaire, ASC—Altered Scales of Consciousness Questionnaire, with subscales: Unity—Experience of Unity, Spiritual—Spiritual Experience, Blissful—Blissful State, Impaired—Impaired Cognition and Control. * *p* < 0.05, ** *p* < 0.01.

**Table 8:** Baseline anxiety and wellbeing (TP1) are associated with typology of acute experience (TP3).

	High Mystical/Low Challenging (*n* = 93)	Low Mystical/High Challenging (*n* = 54)	High Mystical/High Challenging (*n* = 94)	Low Mystical/Low Challenging (*n* = 110)	F	*p*
QIDS_1	5.65	6.44	6.47	5.77	0.927	0.428
STAI_T_1	10.98	12.56	12.68	11.77	3.833	0.010
WEMWBS_1	51.97	47.17	48.77	49.56	4.602	0.007

Key: QIDS_1—Quick Inventory of Depressive Symptoms (Timepoint 1), STAI-T_1—State-Trait Anxiety Inventory (Trait Subscale; Timepoint 1), WEMWBS_1—Warwick-Edinburgh Mental Wellbeing Scale (Timepoint 1).

**Table 9:** Relational triad factors (TP1) are associated with typology of acute experience (TP3).

	High Mystical/Low Challenging (*n* = 93)	Low Mystical/High Challenging (*n* = 54)	High Mystical/High Challenging (*n* = 94)	Low Mystical/Low Challenging (*n* = 110)	F	*p*
SCS	35.33	31.66	33.11	35.11	2.225	0.085
CAMS-R	34.91	30.94	32.25	33.42	6.591	<0.001
Spir_Comp	0.365	−0.221	0.224	−0.273	10.853	<0.001

Key: SCS—Social Connectedness Scale, CAMS-R—Cognitive and Affective Mindfulness Scale-Revised, Spir_Comp—Spirituality Compound Score (*z*-score).

## Data Availability

Data files are available upon request from corresponding author, Joshua Lipson, at joshua.lipson@emory.edu.
